# Idiopathic Normal Pressure Hydrocephalus With Multiple Sulcus Enlargements but No Ventricular Enlargement Resembling Brain Atrophy Over a Long Period Before Diagnosis: A Case Report

**DOI:** 10.7759/cureus.49757

**Published:** 2023-11-30

**Authors:** Shinya Watanabe, Yasushi Shibata, Eiichi Ishikawa

**Affiliations:** 1 Neurosurgery, University of Tsukuba, Tsukuba, JPN; 2 Neurosurgery, Mito General Hospital, Mito, JPN; 3 Neurosurgery, University of Tsukuba Hospital, Tsukuba, JPN

**Keywords:** brain atrophy, occipitoparietal sulcus, enlarged ventricles, desh, idiopathic normal pressure hydrocephalus

## Abstract

The most common imaging findings in idiopathic normal pressure hydrocephalus (iNPH) are disproportionately enlarged subarachnoid space hydrocephalus, i.e., enlarged ventricles (Evans index >0.3), narrowing of the superior arcuate and median sulci, widening of the Sylvian fissure, and focal widening of the sulci of the brain. In the present study, we encountered an interesting case of a 73-year-old woman with iNPH with characteristic imaging findings of cerebral atrophy-like features and no prominent ventricular enlargement. Many cases might be difficult to diagnose as iNPH because of atypical imaging findings such as no prominent ventricular enlargement and so on. Even in cases with multiple atrophic sulcus openings without any prominent ventricular enlargement, the callosal angle and posterior commissure-level brain-ventricle ratio (BVR) could be helpful in the diagnosis, and bilateral opening of the occipitoparietal sulcus might be a key imaging finding.

## Introduction

Idiopathic normal pressure hydrocephalus (iNPH) is characterized on imaging by disproportionately enlarged subarachnoid-space hydrocephalus (DESH) [1], i.e., the coexistence of ventricular enlargement and narrowing of the sulcus and subarachnoid space in the high and middle arcuate regions, as well as widening of the Sylvian fissure [2-5] and focal widening of the sulci [3]. Other indicators include an increase in callosal angle [6] and brain-ventricle ratio (BVR) [7]. However, many iNPH cases with atypical imaging findings might be potentially overlooked. In this study, we present a case of iNPH with brain atrophy-like imaging findings observed over a long period without the typical imaging findings of iNPH.

## Case presentation

A 73-year-old woman developed postural maintenance function deterioration. Six years before visiting our institution, the protrusion phenomenon appeared and progressed. Specifically, symptoms such as leaning forward when standing or running too fast and not being able to stop appeared and progressed. Although she visited several hospitals and clinics, doctors explained that it was caused by brain atrophy. So, she was saddened by the progression of her gait disturbance and at a loss, and she had no choice but to wait and see what would happen. She developed a tendency to fall five years before presentation, and consequently, she experienced a left shoulder dislocation fracture four years before presentation and a right humerus fracture three years before presentation. Her gait disturbance progressed gradually, and she required a cane to walk. Specifically, she was no longer able to walk without a cane and slow, careful steps. She visited the neurologist at our hospital. The Mini-Mental State Examination (MMSE) score was 30 points, and no dysuria was detected. Her gait disturbance improved with a tap test, and she was referred to our department. She did not smoke or drink alcohol, and she had no allergies. She was married after graduating from high school, had a child, and lived peacefully as a housewife of gentle disposition. Her medical history included uterine fibroid surgery at age 42, stage Ⅲb right breast cancer that went into remission after surgery followed by chemotherapy at age 59, and dyslipidemia requiring statin therapy at age 70. Her family history was not significant.

Physical examination revealed a small stature with a height of 148 cm, a weight of 51 kg, and a body mass index of 23.3. Regarding awareness and higher functions, her score was 15 (4-5-6) points on the Glasgow Coma Scale and 30/30 points on the MMSE. In the cranial nervous system, there were no visual field abnormalities, oculomotor disturbances, contra lesional reflexes (+/+), facial paralysis, or dysarthria. Assessment of the motor system revealed a negative Barre’s sign in both the upper and lower limbs, a grade of 5 on the Manual Motor Test in both the upper and lower limbs, and grip strengths of 25 and 23 kg in the right and left hands, respectively. No obvious sensory disturbances were observed in the whole body. The coordination of hand rotation was smooth. Blood testing revealed no obvious abnormality.

**Figure 1:**
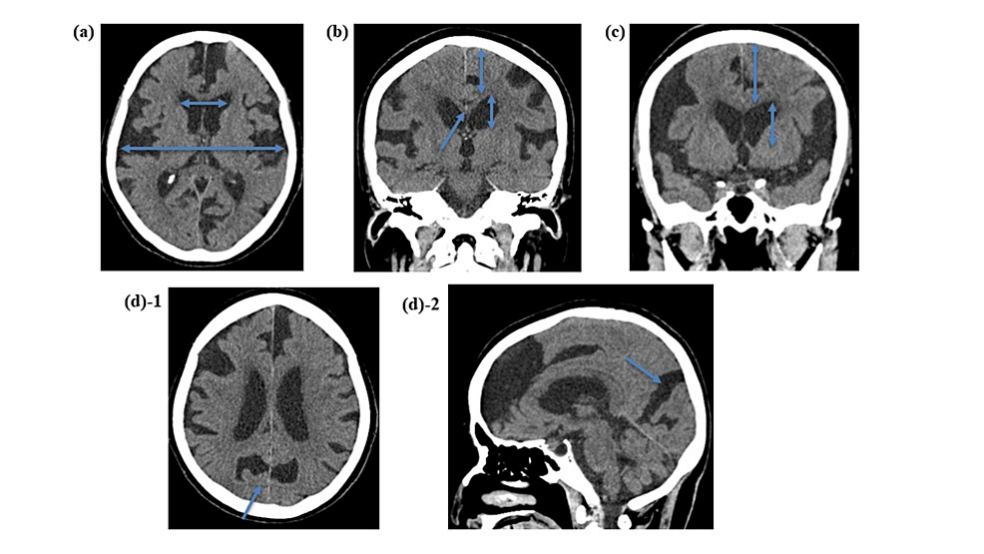
Computed tomography images of the patient experiencing gait disturbance. (a) An axial image showed an Evans index of 0.29 (blue two-headed arrows). (b) A coronal image of PC level showed diffuse cerebral sulcus enlargement, a tendency toward widening of the Sylvian fissure and narrowing of the median sulcus and subarachnoid space, a callosal angle of <90° (a blue arrow), and a PC-level BVR of 1.46 (blue two-headed arrows). (c) A coronal image of AC level showed BVR of 1.11 (blue two-headed arrows). (d)-1. Axial images showed bilateral enlargement of the occipitoparietal sulcus (a blue arrow). (d)-2. A Sagittal image of bilateral enlargement of the occipitoparietal sulcus (a blue arrow). PC: posterior commissure, BVR: brain–ventricle ratio, AC: anterior commissure.

Head computed tomography (CT, Figure 1) revealed an Evans index of 0.29. The Evans index of 0.30 or higher, the so-called definition of ventricular enlargement, was not met, and therefore, there was no obvious marked ventricular enlargement. Diffuse, rather than focal, cerebral sulcus enlargement was noted, along with a generalized cerebral atrophy-like appearance. The examination of not only axial images but also coronal and sagittal images revealed a tendency toward widening of the Sylvian fissure and narrowing of the median sulcus and subarachnoid space. In addition, the imaging findings included a callosal angle [6] of <90° (positive criterion, <90°), posterior commissure (PC)-level BVR of 1.46 (positive criterion, <1.5), anterior commissure-level BVR [7] of 1.11 (positive criterion, <1.0), and bilateral enlargement of the occipitoparietal sulcus.

In the tap test, the initial lumbar puncture pressure was 20 cmH_2_O, meeting the criterion for normal pressure hydrocephalus [8], and laboratory data from the cerebrospinal fluid (CSF) sample included a glucose level of 58 mg/dL, a chloride level of 125 mEq/L, a protein level of 41 mg/dL, and a cell count of 1/3 (1 mononuclear cell, 0 multinuclear cells) (Table 1).

**Table 1:** Laboratory data from the cerebrospinal fluid sample.

	Value	Normal range
Glucose	58 mg/dL	40–75
Chloride	125 mEq/L	120–125
Protein	41 mg/dL	10–40
Cell count	1/3	0–5

**Figure 2:**
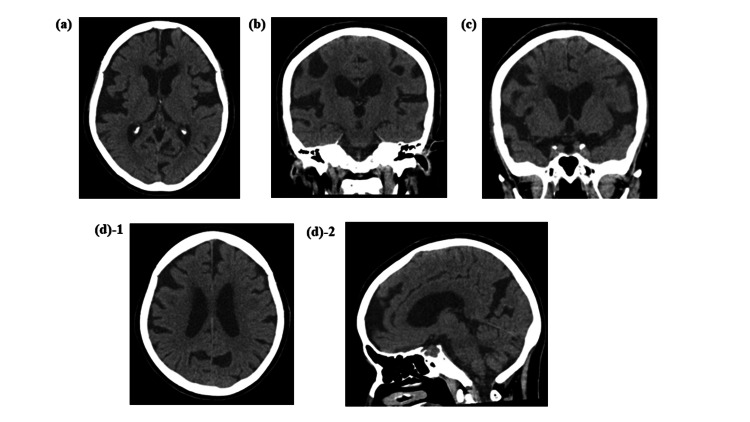
CT images at five post-shunting months.

The result of the Timed Up and Go test before the tap test was 10.1 seconds on 13.7 steps, the MMSE score was 30/30 points, and the Frontal Assessment Battery score was 13/18 points, which improved to 9.5 seconds on 13.7 steps, 30/30 points, and 16/18 points, respectively. In the assessment of these findings, head CT revealed no ventricular enlargement, many sulci were open, and a cerebral atrophy-like appearance was noted. However, the Sylvian fissure was widened, the median sulcus and subarachnoid space were narrowed, and her gait disturbance was improved after the tap test. Therefore, the patient was considered suitable for shunt surgery because of her symptomatic improvement on the tap test. A lumbo-peritoneal shunt procedure was performed as molded. Post-shunting CT images (Figure 2) showed improvement in the narrowing of the median structures, the opening of the cerebral sulcus, subarachnoid space, Sylvian fissure, and the parieto-occipital sulcus as well.

The postoperative course was good with an objective improvement in gait disturbance (Table 2), and the patient has been able to walk without a cane since discharge.

**Table 2:** Evaluation before and after shunting TUG: Timed Up and Go test; MMSE: Mini-Mental State Examination; FAB: Frontal Assessment Battery

	Preshunting	Postshunting	5 months postshunting
TUG	9.8 s	10.8 s	8.0 s
10-meter walk	13.5 s 22 steps	8.4 s 15 steps	8.2 s 16 steps
MMSE	30/30	30/30	-
FAB	12/18	14/18	-

## Discussion

We have described a case of iNPH with atypical imaging findings in which the presence of hydrocephalic pathology was not diagnosed because of the absence of prominent ventricular enlargement, numerous open cerebral sulci, and a cerebral atrophy-like appearance, and the patient suffered and was not treated as iNPH for a long period of time.

What other findings should make you suspect iNPH even in the absence of ventricular enlargement?

DESH is a characteristic imaging finding of iNPH, but visual assessment is sometimes difficult. It has been reported that DESH findings have a positive predictive value of 77% but a low negative predictive value of 25% [9]. In the present case, the DESH features of coexisting narrowing of the median sulcus and subarachnoid space and widening of the Sylvian fissure were observed, but the multiple sulcus openings made it difficult to distinguish. The callosal angle [6] and PC-level BVR [7] were helpful and characteristic, as was the finding of bilateral opening of the occipitoparietal sulcus. Even in cases in which DESH is difficult to identify, these callosal angle and PC-level BVR findings can be helpful to diagnose. Furthermore, the importance of artificial intelligence (AI) in neurosurgery has increased significantly over the past decade; AI can improve diagnostic and prognostic outcomes in clinical care, assist neurosurgeons in decision-making during surgical interventions to improve patient outcomes, and provide the best interventional and non-interventional care to patients. It can complement the neurosurgeon's skills in providing the best interventional and non-interventional care to patients and offer great potential for neurosurgery [10]. We believe it is important to accumulate characteristic findings, including bilateral enlargement of the parieto-occipital sulcus in cases with inconspicuous ventricular enlargement findings, for future diagnostic use of AI.

How many similar cases have been reported and how many cases have been overlooked?

The incidence of iNPH in the Japanese population has been reported in previous studies to be approximately 1,200/100,000 persons/year [11]. However, the number of hospital visits for patients with iNPH is estimated to be 30-60 per 100,000 people per year [12,13]. The incidence of gait disturbance was reported to be 94-100% in almost all studies, which was consistent with most iNPH patients. Cognitive and urinary disturbances were reported to be 78-98% and 60-92%, respectively [8]. Therefore, it is estimated that at least a few percent of patients have only gait disturbance as a symptom. There are obstacles to visits attributable to the characteristics of the disease; the most common symptom, gait disturbance, is characterized by gradual progression, a variety of causative diseases, and difficulty in diagnosis, but surprisingly, in the present case, the patient was under observation after visiting several departments and hospitals for more than five years because of gait disturbance. The challenge for primary care physicians is identifying imaging findings that can serve as an easily understood surrogate for the presence of iNPH, as DESH is difficult to detect in such patients because of the lack of ventricular enlargement.

Was focal sulcus enlargement a feature of this case and where has this been reported in the literature?

Some imaging parameters have been described for iNPH with ventricular enlargement. Kuchcinski et al. reported that the best parameter for discriminating patients with iNPH with ventricular enlargement from those with vascular cognitive impairment and healthy controls was the calcarine sulcus-cingulate sulcus aperture ratio with an area under the curve of 0.94 (95% confidence interval = 0.89-0.99). The cutoff of 0.95 had the highest sensitivity (96.8%) and specificity (83.3%) [14]. However, no report on imaging parameters for iNPH without ventricular enlargement has been published, and we hope that this case will be helpful and will deepen our knowledge. In this case, bilateral enlargement of the parieto-occipital sulcus was characteristically observed, which may be a trigger for differentiating iNPH without ventricular enlargement, and we expect to accumulate more cases in the future. To achieve this goal, it will be necessary to launch working groups, activate clinical research on iNPH, which lacks ventricular enlargement, and eventually educate medical practitioners.

Why was there no ventricular enlargement and why did iNPH symptoms still occur?

Although the fourth ventricle and subarachnoid space of the alveolar region are connected by the foramina of Luschka and Magendie in healthy subjects, it was recently suggested that the fourth ventricle and subarachnoid space are connected by the choroid plexus [15]; the most likely site of connection is the choroid plexus, in which the ventricles and subarachnoid space are separated by a thin membrane called the tenia at the base of the choroid plexus attachment. In particular, the inferior choroidal point of the choroidal fissure was supposed to be an arteriovenous pathway that serves the diverticular canal and inferior horn of the lateral ventricle, and the tenia of the fimbria is easily connected by extension [16]. As the CSF volume increases, these temporary CSF channels open, causing the lateral ventricles and basilar and Sylvian fissures to expand in unison and push upward in a parietal direction, compressing the brain and arachnoid cavity in the high circumflex area and resulting in DESH [15]. It has been reported that the density of the arachnoid columns lining the basilar and Sylvian fissures can determine whether the ventricles or the basilar and Sylvian fissures are more likely to expand [16]. Based on these reports, it can be concluded that the density of the arachnoid column might be low in this case. In atypical cases of imaging, such as no ventricular enlargement, many sulci are open and have the possibility of being iNPH, so it is important to perform a tap test to identify undiagnosed cases of hydrocephalus.

## Conclusions

Even in cases without ventricular enlargement and with a cerebral atrophy-like appearance and multiple sulcus openings, the tendency of the median sulcus and subarachnoid space to narrow is a reliable indicator of iNPH, and the callosal angle and PC-level BVR might be helpful. In addition, the bilateral opening of the occipitoparietal sulcus could provide a clue to the imaging findings. We believe that these findings might help undiagnosed iNPH without ventricular enlargement to be performed by a tap test.
